# Engineering 2,4,6‐Triaminopyrimidine‐Functionalized Fumed Silica for Highly Selective Hg^2+^ Detection: Spectroscopic and Theoretical Elucidations

**DOI:** 10.1002/open.70257

**Published:** 2026-07-17

**Authors:** Ghodsi Mohammadi Ziarani, Fatemeh Soleymani, Zahra Panahande, Zahra Ahmadi, Alireza Badiei, Senem Akkoc, Mehran Feizi‐Dehnayebi

**Affiliations:** ^1^ Department of Organic Chemistry Faculty of Chemistry Alzahra University Tehran Iran; ^2^ School of Chemistry College of Science University of Tehran Tehran Iran; ^3^ Department of Basic Pharmaceutical Sciences Faculty of Pharmacy Suleyman Demirel University Isparta Türkiye; ^4^ Faculty of Engineering and Natural Sciences Bahcesehir University Istanbul Türkiye

**Keywords:** density functional theory (DFT), fluorescence, fumed‐silica, functionalization, Hg^2+^ ions

## Abstract

Hg^2+^ ions are highly toxic, persistent, and bioaccumulative heavy metal pollutants that pose a serious threat to human health, making the development of highly sensitive and selective chemosensors for their detection essential. In this research, a new hybrid material, fumed‐Si‐Pr‐TAP, was prepared by stepwise functionalization of fumed‐silica with 3‐(chloropropyl)trimethoxysilane and 2,4,6‐triaminopyrimidine (TAP). The prepared material was fully characterized using various techniques, including Fourier transform infrared (FT‐IR), thermogravimetric analysis (TGA), N_2_ adsorption–desorption analysis, scanning electron microscopy (SEM), and energy dispersive X‐ray spectrometry (EDX). The sensing studies revealed that this material can detect Hg^2+^ ions with good selectivity and an acceptable detection limit of 3 × 10^−7^ M in ethanolic media. This improved performance is attributed to the presence of nitrogen‐rich TAP moieties on the fumed silica surface, which act as highly efficient coordination sites, making this hybrid compound outstanding for advanced chemosensor applications. Density functional theory calculations were carried out to better understand the interaction between the Pr‐TAP ligand and Hg^2+^ ions at the molecular level. The optimized structures and electrostatic analyses reveal that nitrogen‐rich regions serve as the primary binding sites, supporting strong coordination with mercury. Changes in the electronic structure, particularly the reduced HOMO–LUMO gap upon complex formation, highlight an increase in chemical reactivity that underpins the sensing behavior.

## Graphical abstract

Hg^2+^ is considered one of the most dangerous heavy metal pollutants. The fumed‐Si‐Pr‐TAP was prepared by functionalizing fumed‐silica with 3‐(chloropropyl)trimethoxysiliane and 2,4,6‐triaminopyrimidine (TAP). Fumed‐Si‐Pr‐TAP can detect Hg^2+^ ions in aqueous solutions with good sensitivity and selectivity. Density functional theory calculations were carried out to better understand the interaction between the Pr‐TAP ligand and Hg^2+^ ions at the molecular level. Changes in the electronic structure, particularly the reduced HOMO–LUMO gap upon complex formation, highlight an increase in chemical reactivity that underpins the sensing behavior.

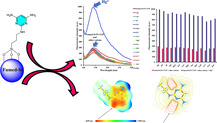

## 1 Introduction

Currently, due to the rapid growth of urban, industrial, agricultural, and transportation activities, especially in coastal areas, heavy metals are entering coastal and marine environments in large quantities through industrial effluents [1]. Since these metals remain in the environment for a long time due to their persistence and nonbiodegradability, they gradually put great pressure on aquatic ecosystems and pose a serious threat to human and the environmental sustainability. Marine sediments act as the main reservoir of metals transported through rivers and atmosphere, because these metals tend to attach to the suspending particles [2, 4]. Heavy metals, with high atomic weight and a density 5 times higher than water, are highly toxic. These heavy metal ions, containing, Pb^2+^, Cd^2+^, As^3+^, Cr^3+^, and Hg^2+^, may cause serious diseases, including acute or chronic poisoning, when exposed to food, air, and water above their safe thresholds and persist in aquatic environments for long periods [5]. These elements not only accumulate in sediments but also enter the bodies of living organisms, causing a variety of biological effects, from cell function to damage to growth and reproduction [6, 9]. These are transferred through the process of bioaccumulation and bioconcentration along food chains. For this reason, consumption of seafood is one of the most important routes of human exposure to these metals, and it has become a public health concern worldwide [10, 14]. It exists in forms such as elemental mercury (Hg^0^), inorganic mercury (I‐Hg), and organic mercury, such as methylmercury (MeHg), each with distinct environmental behavior and toxicity [15, 17]. Given the evidence of the harmful effects of mercury on human health, it is of great importance to review standards safely, maintain regulatory laws, improve systems, and increase public information. At the same time, the application of new advanced biofiltration purification methods and technologies can play an important role in reducing the environmental impacts of mercury and protecting public health [18, 21]. In the field of detection, optical methods based on fluorescence change have become practical and reliable tools for the measurement of Hg^2+^ ions due to their simplicity, high response speed, and suitable sensitivity [22, 23]. Meanwhile, systems based on fluorescence probes, which reveal the presence of the analyte through measurable changes in intensity or emission wavelength, have made significant progress in recent years, enabling rapid and sensitive identification [24, 31]. Although classical methods such as inductively coupled plasma mass spectroscopy (ICP‐MS) [32, 33] and atomic absorption spectroscopy (AAS) [34] have very high accuracy and very low detection limits, the need for expensive equipment, skilled labor, and complex sample preparation limits their use for rapid and field measurements [35]. The development of functionalized nanomaterial that possess Hg (II) adsorption sites is of great importance because these materials can effectively adsorb Hg^2+^ ions through soft–soft interactions. The nitrogen‐rich organic groups placed on the silica surface act as effective mercury binding sites, making these materials efficient adsorbents for Hg^2+^ removal [36]. Fumed silica, also known as pyrogenic silica, is an amorphous silicic acid, which is usually produced by the hydrolysis and flame pyrolysis of silicon tetrachloride or the evaporation of quartz sand at very high temperature in an electric arc furnace. It has a significant surface activity and reactivity due to its high specific surface activity and the presence of silanol (OH) groups on the surface. The particle size in the nanoscale design, three‐dimensional, nonporous, and contact surface make fumed silica an efficient material for applications such as pollutant adsorption, biosensor, and elutriation systems. In addition, nanomaterials and composites in general have attracted much attention in the field of sensing technologies due to their higher levels of bulk density, tunability of porous structure, and desirable electronic properties, as these properties contribute to increasing and improving the efficiency of diagnostic systems [37, 40]. Following our studies on the preparation of different chemosensors [41, 43], herein, a new chemosensor has been synthesized through the functionalization of fumed silica with 2,4,6‐triaminopyrimidine (TAP).

## 2 Materials and Analytical Methodologies

All materials and solvents used in this study were obtained from Merck. In order to identify the structure and investigate the physicochemical properties of the samples, various analytical techniques were used. Thermogravimetric analysis (TGA) spectroscopy was performed using a BAHR Thermoanalyse (STA 503) instrument. Fourier transform infrared (FT‐IR) spectroscopy was accomplished with a Bruker Tensor 27 using KBr tables. The surface morphology of the samples was examined using a TESCAN MIRA3 scanning electron microscope (SEM). Also, a combination of energy dispersive X‐ray spectrometry (EDX) components was determined with a VEGA TESCAN‐LMU device.

### 2.1 Experimental Part

#### 2.1.1 The Synthesis of Fumed‐Si‐Pr‐Cl

Fumed‐silica (1 g) was dried and activated in an oven at 100 °C for 2 h. Then, 3‐(Chloropropyl)trimethoxysilane (3 mL) was added, and it was refluxed in toluene (50 mL) for 5 days. Lastly, the gained solid was purified by centrifugation with EtOH and acetone, and then dried.

#### 2.1.2 The Synthesis of Fumed‐Si‐Pr‐TAP

Fumed‐Si‐Pr‐Cl (1 g) was placed in an oven to dry and activate at 100 °C for 2 h. Then, TAP (1 g), Et_3_N (1.5 mL), and toluene (50 mL) were added to it and then refluxed for 5 days. The resulting precipitate was centrifuged, followed by washing with EtOH, acetone and then dried.

### 2.2 Computational Perspective

A computational study was carried out using the GaussView 6.0/Gaussian 09 W package [44] to support and rationalize the interaction mechanism proposed from the experimental observations. Geometry optimizations were performed within the framework of density functional theory (DFT) employing the B3LYP functional. In these calculations, the LANL2DZ basis set was assigned to Hg^2+^, while the 6‐311 g (d) basis set was applied to the remaining atoms (H, N, and C). The most probable binding region toward the metal ion was located by analyzing the molecular electrostatic potential (MEP) and electrostatic surface potential (ESP) maps. Chemical reactivity was further interpreted through molecular orbital analysis.

## 3 Results and Discussion

**SCHEME 1:**
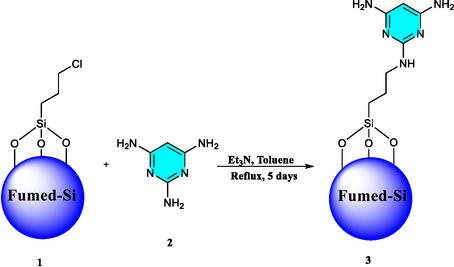
Synthesis of fumed‐Si‐Pr‐TAP.

Initially, the fumed‐silica surface was functionalized with 3‐(chloropropyl)trimethoxysiliane, leading to the preparation of fumed‐Si‐Pr‐Cl, which was then functionalized with TAP to form fumed‐Si‐Pr‐TAP as a new heterogeneous material (Scheme 1).

### 3.1 Characterization of Fumed‐Si‐Pr‐TAP

#### 3.1.1 FT‐IR

**FIGURE 1:**
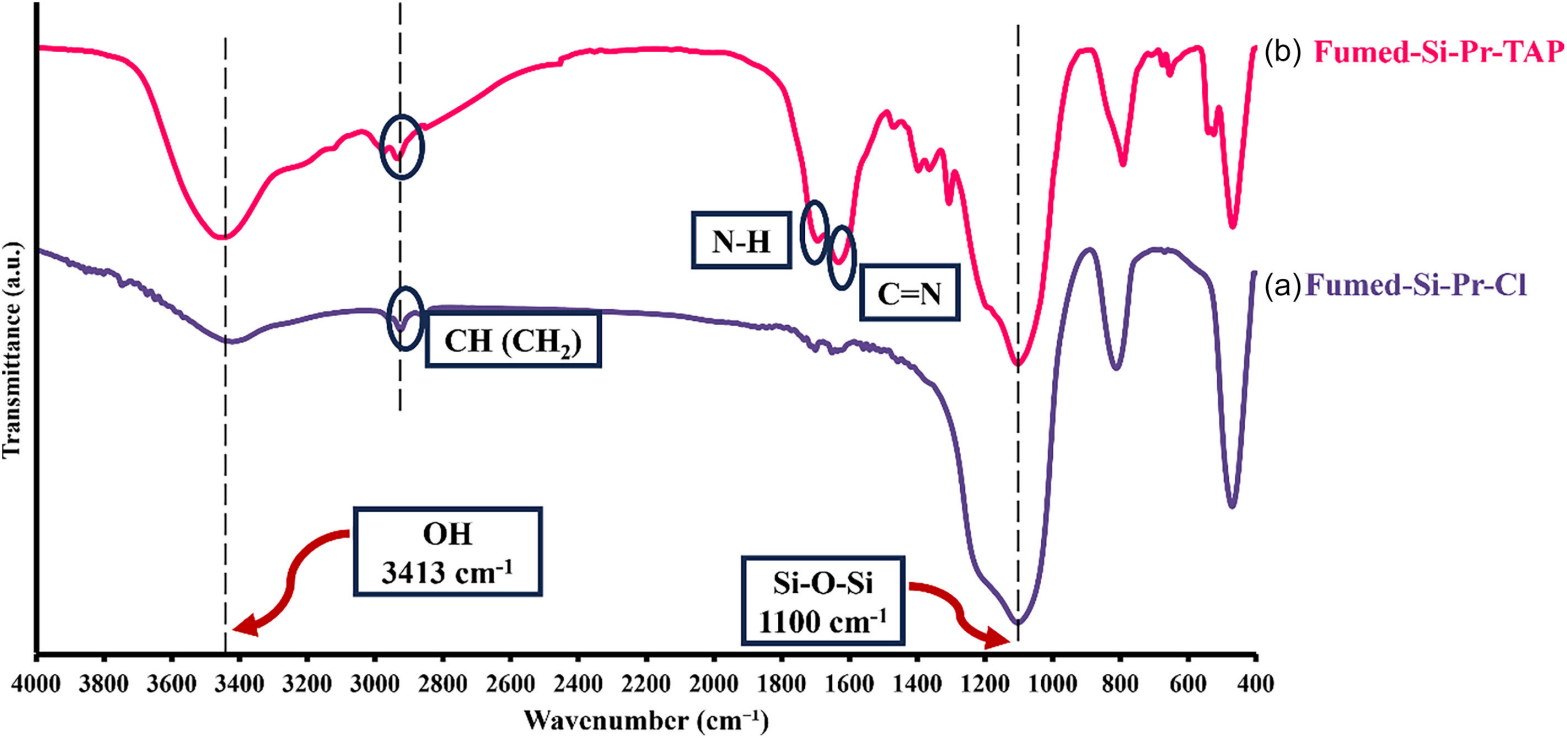
FT‐IR spectra for (a) fumed‐Si‐Pr‐Cl and (b) fumed‐Si‐Pr‐TAP.

The FT‐IR spectra for two compounds fumed‐Si‐Pr‐Cl and fumed‐Si‐Pr‐TAP, are demonstrated in Figure 1. In samples a and b, absorption bands at 1100 and 3413 are correspondingly related to the Si–O–Si and Si–OH vibrations in fumed‐silica. Also, the peak at 2923 cm^−1^ can be attributed to the C–H in propyl chains. In the spectrum (b), the absorption bands in the regions 1629 and 1691 cm^−1^ are correlated to the vibrations of the TAP C=N and N–H groups, which confirms the placement of the TAP group on the fumed‐silica.

#### 3.1.2 TGA Analysis

**FIGURE 2:**
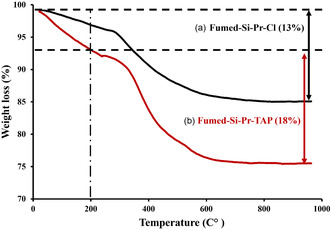
TGA analysis for samples.

The TGA plot demonstrated that both samples show a slight weight loss at low temperatures, up to about 150–200 °C, due to the removal of moisture and adsorbed surface groups, but the fumed‐Si‐Pr‐TAP samples show a sharper weight loss than the fumed‐Si‐Pr‐Cl. This sharper weight loss is attributed to the higher hydrophilicity of fumed‐Si‐Pr‐TAP, resulting from the introduction of polar nitrogen‐rich groups (amine and pyrimidine moieties) on the surface. In contrast, fumed‐Si‐Pr‐Cl exhibits more hydrophobic character due to the presence of relatively nonpolar chloropropyl groups. The total weight loss is reported to be about 13% for fumed‐Si‐Pr‐Cl and about 18% for fumed‐Si‐Pr‐TAP, indicating an increase in the amounts of organic groups attached to the surface after TAP modification (Figure 2). The major weight loss in the range of 300–600 °C is related to the thermal decomposition of organic chains attached to the silica surface. Therefore, the increased weight loss percentage in the TAP sample indicates a more successful functionalization and a higher load of organic groups on the silica surface. In contrast, the high‐weight residue at the final temperatures indicates the inorganic stability of the silica.

#### 3.1.3 SEM and EDX Analysis

**FIGURE 3:**
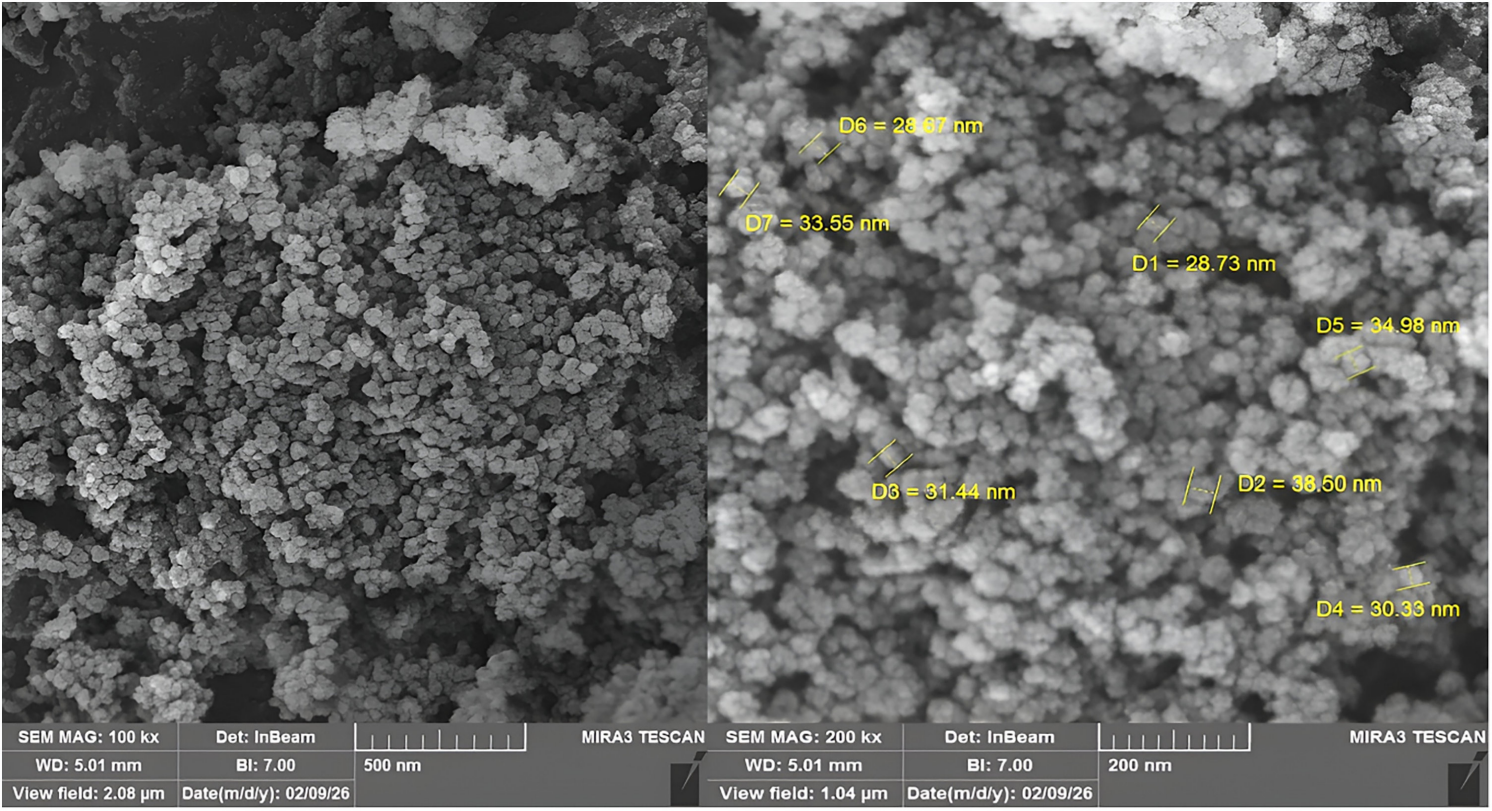
SEM images for fumed‐Si‐Pr‐TAP.

**FIGURE 4:**
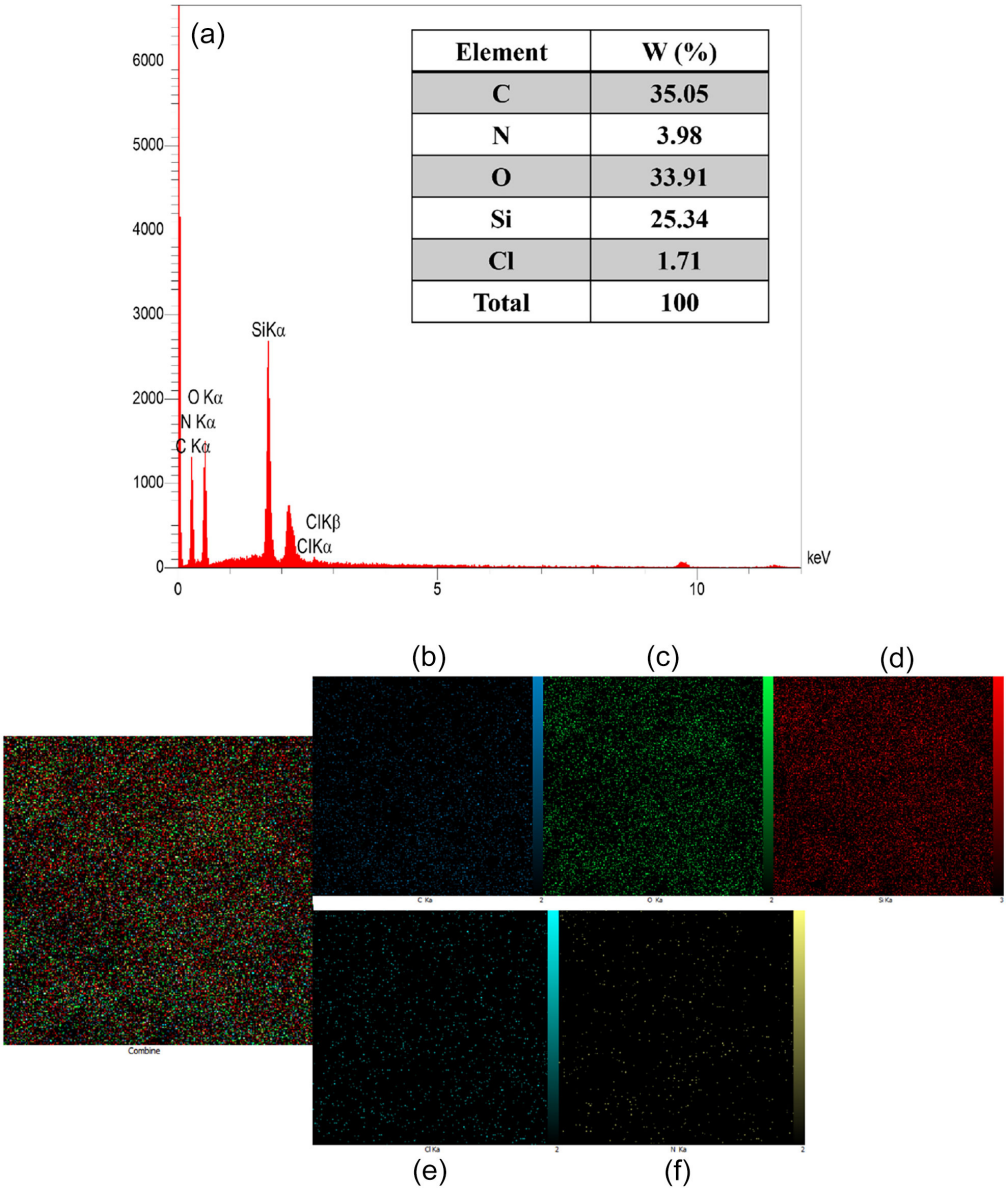
EDX analysis for fumed‐Si‐Pr‐TAP (a) and color mapping: overall mapping of carbon (b), oxygen (c), silica (d), chlorine (e), and nitrogen (f).

SEM is an advanced analytical technique for studying the morphology and composition of material surfaces. In this technique, valuable information about the surface texture, topology, and structural features of the material is obtained by scanning the sample surface with an electron beam and forming an image. Figure 3 demonstrates SEM images of the modified fumed‐Si‐Pr‐TAP sample, which are used for morphological evaluation. These images show that after surface modification, the overall structure, including spherical particles, pores, and channels of fumed‐silica, does not change significantly. In addition, EDX measurements were performed to determine the components of the fumed‐Si‐Pr‐TAP sample, which showed the presence of C (35.05), N (3.98), O (33.91), Si (25.34), and Cl (1.71) (Figure 4).

#### 3.1.4 N_2_ Adsorption–Desorption Analysis

**FIGURE 5:**
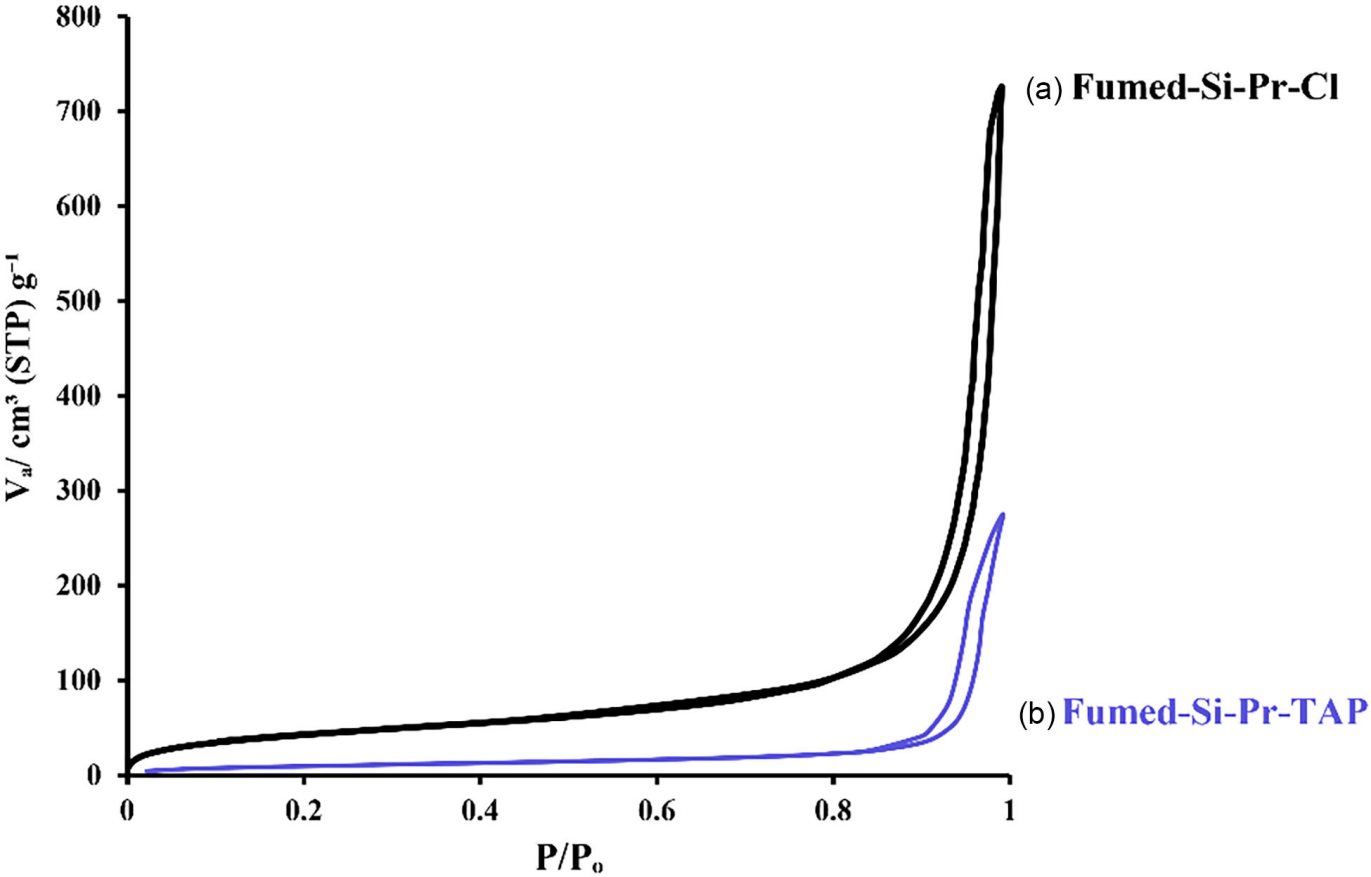
N_2_ adsorption–desorption isotherms.

N_2_ adsorption–desorption isotherms are demonstrated in Figure 5 for fumed‐silica‐Pr‐Cl and fumed‐silica‐Pr‐TAP, which indicate the aggregate nature and interparticle spaces in the structure of fumed‐silica. Furthermore, Table 1 shows that after modification of the surface of fumed‐silica‐Pr‐Cl with TAP groups, the amount of nitrogen adsorption decreased, which is in good agreement with the sharp decrease in BET specific area, indicating effective coverage of the silica surface by organic groups. In contrast, the total pore volume does not change significantly, indicating the relative preservation of interparticle spaces along with the reduction of surface accessibility. Also, the slight increase in the average diameter is attributed to the change in the particle aggregation arrangement and the relative increase in interparticle spaces. Overall, the isotherm results and BET specific area well confirm the success of the surface modification process.

**TABLE 1:** Textural properties of fumed‐Si‐Pr‐Cl and fumed‐Si‐Pr‐TAP.

Sample	***S***_**BET**_, **m** ^**2**^ **g** ^**−1**^	***D***_**nm**_	***V*, cm** ^**3**^ **g** ^**−1**^
Fumed‐Si‐Pr‐Cl	157.6	23	0.38
Fumed‐Si‐Pr‐TAP	34.3	29	0.41

### 3.2 Fluorescence Activity

#### 3.2.1 Detection of Hg^2+^ Ions and Selectivity Test

**FIGURE 6:**
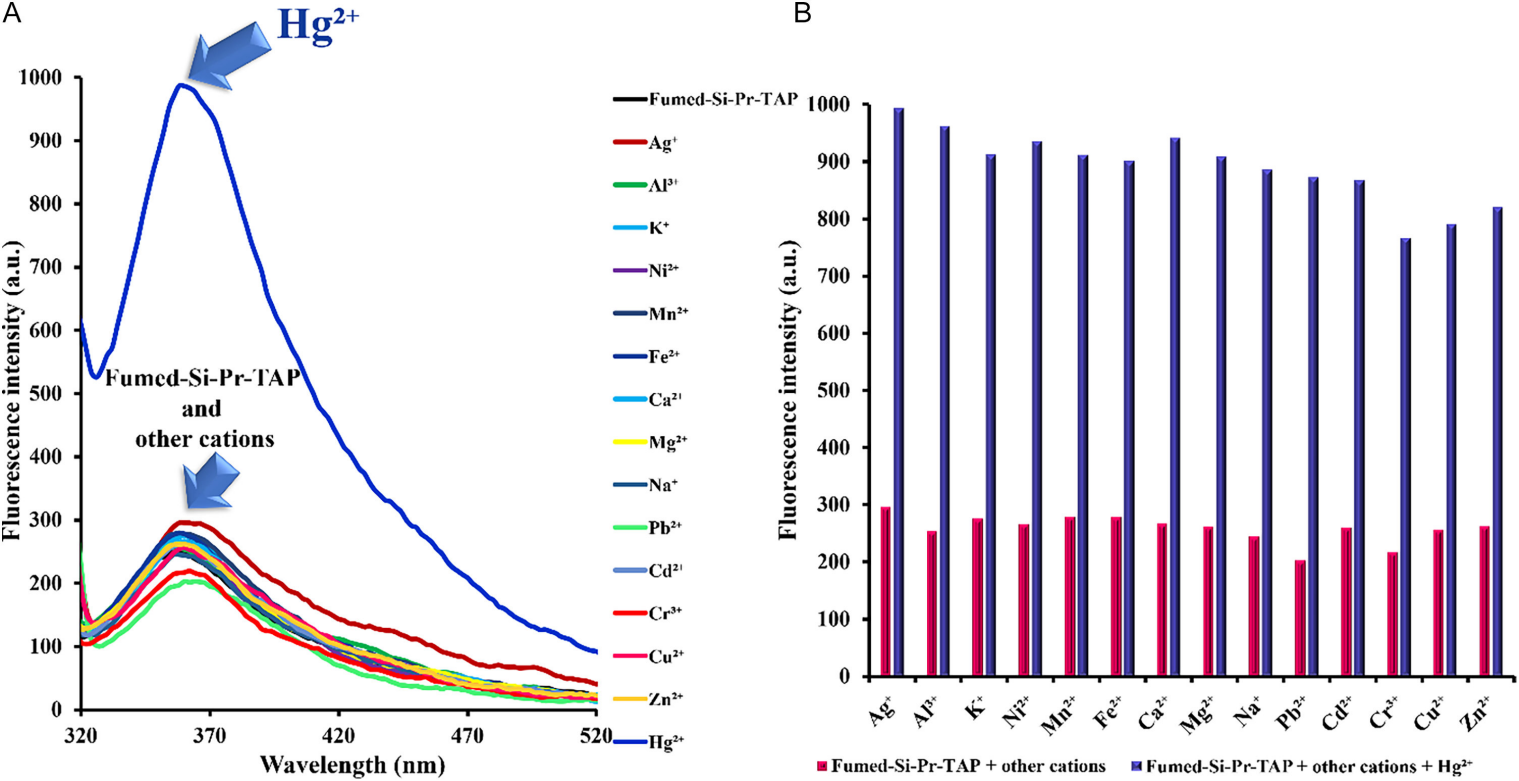
(A) Fluorescence response of fumed‐Si‐Pr‐TAP (2.5 mL, 0.2 g/L^−1^) after the addition of various cations (λ_ex_ = 300 nm, λ_em_ = 360 nm) and (B) competitive test for sensing Hg^2+^ ions.

A 0.01 M solution of nitrate salts of different metal ions, including Ag^+^, K^+^, Na^+^, Mn^2+^, Ni^2+^, Fe^2+^, Mg^2+^, Zn^2+^, Hg^2+^, Cu^2+^, Pb^2+^, Ca^2+^, Cd^2+^, Al^3+^, and Cr^3+^, was prepared. Also, 0.02 g of the fumed‐silica‐Pr‐TAP was dispersed in 100 mL of EtOH. Then, 2.5 mL of the fumed‐Si‐Pr‐TAP was evaluated in the presence of 200 µL of various cations, and the emission intensity was recorded at an excitation wavelength of 300 nm. The results (Figure 6A) demonstrated that the fluorescence intensity at 360 nm increased significantly with the addition of 200 µL of Hg^2+^ ions, while other ions did not cause a significant change, indicating a specific response of this system to Hg^2+^ ions. The importance of examining selectivity is to determine that the signal reduction is due to the selective interaction with Hg^2+^ ions and that competing ions do not cause a false response. The competition test (Figure 6B) also showed that the presence of other cations did not inhibit the brightening effect of Hg^2+^ ions, and no significant interference was observed.

#### 3.2.2 Titration Test and LOD Calculation

**FIGURE 7:**
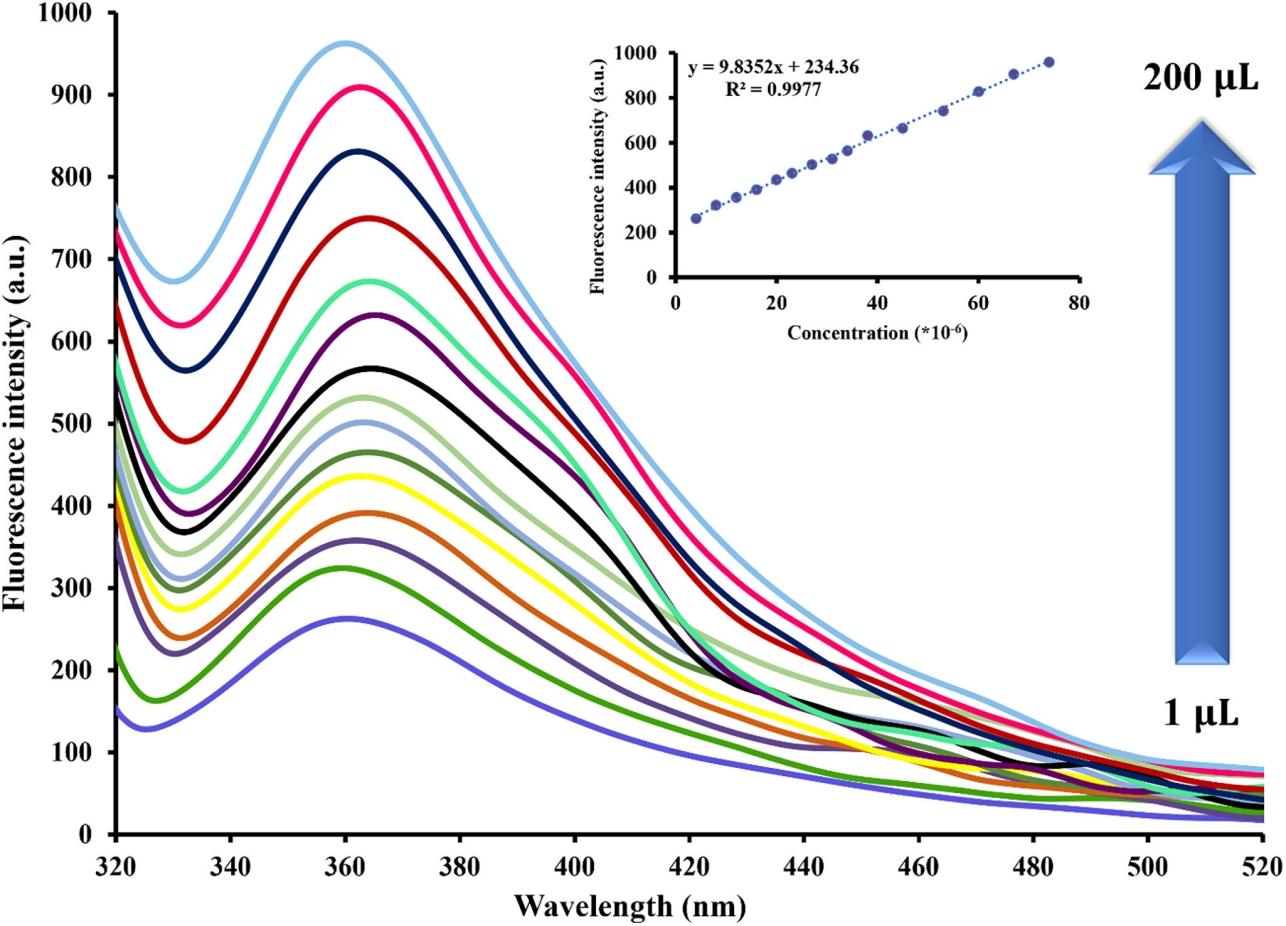
Fluorescence response in the presence of various concentrations of Hg^2+^ ions.

A titration test was performed to estimate the sensitivity of the fumed‐Si‐Pr‐TAP chemosensor to the Hg^2+^ ions (Figure 7) with a gradual increase in the volume from 1 to 200 µL. The fluorescence intensity of the fumed‐Si‐Pr‐TAP probe increased in a concentration‐dependent manner, which demonstrates the effective interaction between the Hg^2+^ ions and the active sites in the nanohybrid structure and the enhancement of the fluorescence emission process. Regular changes in the emission intensity at the various concentrations indicate a quantitative and measurable response of the system to the presence of the analyte. Similarly, by plotting the emission intensity versus Hg^2+^ ion concentration and applying the equation DL = *K* × *S*_d_/*m* (where *K* is 3, *S*_d_ is the standard deviation of the blank sample, and m is the slope of the calibration line), the limit of detection (LOD) was calculated to be 3 × 10^−7^ molar for Hg^2+^ ion, which indicates the desired good sensitivity of this chemosensor in detecting low amounts of Hg^2+^ ions.

#### 3.2.3 Possible Mechanism for Interaction

**FIGURE 8:**
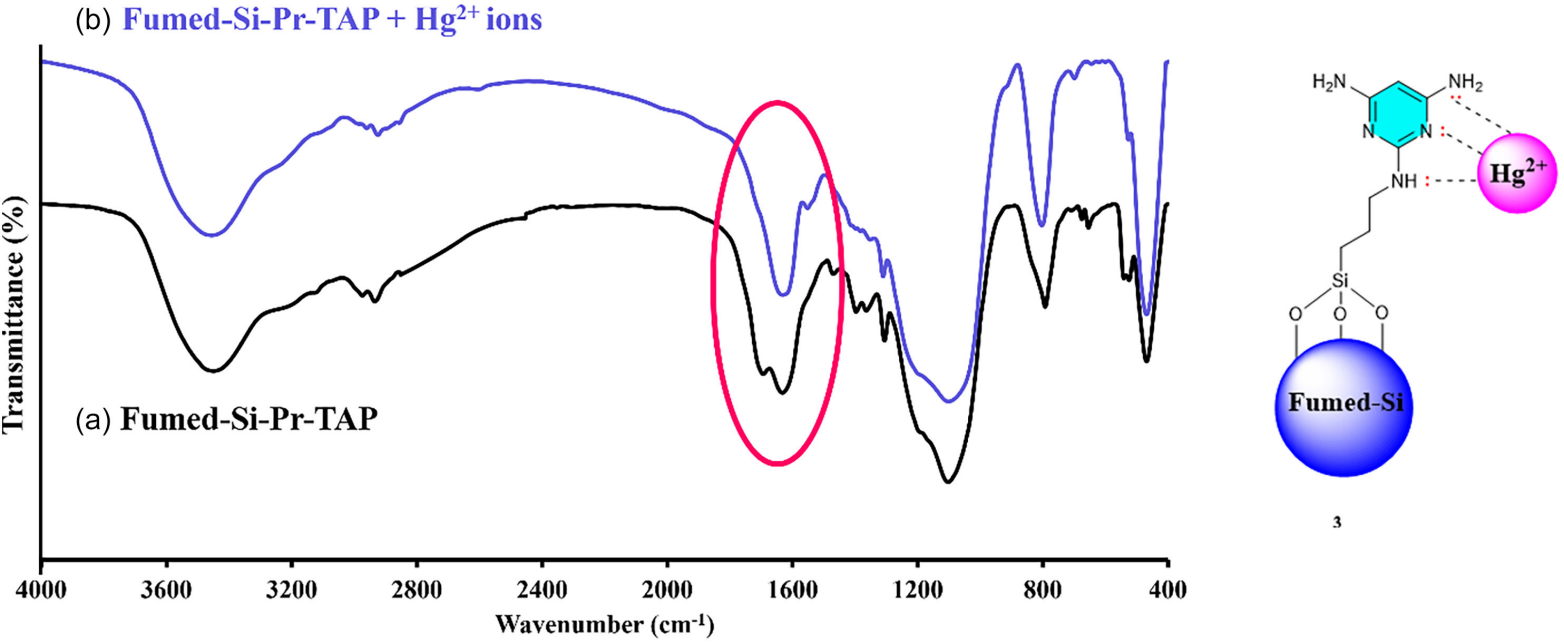
FT‐IR comparison and probable interaction of fumed‐Si‐Pr‐TAP with Hg^2+^ ions.

The comparison of FT‐IR spectra before and after interaction with Hg^2+^ ion is shown in Figure 8. After the addition of Hg^2+^ ion, the intensity of the bands corresponding to the amino groups and nitrogens of the ring, especially in the regions of 1600–1700 cm^−1^, decreased with Hg^2+^ ion. In fact, the nitrogen atoms in fumed‐Si‐Pr‐TAP have lone pairs of electrons and can act as electron donors. On the other hand, the Hg^2+^ ion, due to its soft Lewis acid nature, has a strong tendency to interact with nitrogen centers through the formation of N → Hg coordination bonds. As a result, the observed changes in the FT‐IR spectrum confirm the possible N‐Hg^2+^ binding and provide the basis for the selective detection of Hg^2+^ ions by the nanochemosensor.

#### 3.2.4 Evaluating the Sensing of Fumed‐Pr‐Si‐TAP With Other Chemosensors

A comparative Table 2 has been provided to evaluate the performance of the proposed chemosensor against other chemosensors reported for Hg^2+^ ion detection. As shown in the table, this chemosensor exhibits a reasonably superior detection limit compared to many of the reported systems.

**TABLE 2:** Comparison of fumed‐Pr‐Si‐TAP with other chemosensors.

Entry	Name	LOD	Ref.
1	Fumed‐Si‐Pr‐PNS^a^	12.45 × 10^−6^ M	[42]
2	GO‐SiO_2_‐ABED^b^	2.2 × 10^−4^ M	[45]
3	Fumed‐‍Si‐‍Pr‐‍Ald‐‍Barb^c^	5.4 × 10^−3^ M	[46]
4	Fumed‐‍Si‐‍Pr‐‍N‐‍Ter‐‍Ind^d^	3.68 × 10^−7^ M	[47]
5	Fumed‐Si‐Pr‐TAP^e^	3 × 10^−7^ M	In this work

a Fumed‐Si‐Pr‐PNS: Fumed silica was modified with 3‐(chloropropyl)trimethoxysilane, piperazine and 2‐naphthalenesulfonyl chloride.

b GO‐SiO_2_‐ABED: N1, N1(anthracene‐9,10‐diylbis(methylene)bis(2‐aminoethyl)ethane‐1,2‐diamine) onto GO‐SiO_2_.

c Fumed‐Silica‐Pr‐Ald‐Bar: Fumed silica was modified with 3‐(chloropropyl)trimethoxysilane, pyridine carbaldehyde, and barbituric acid.

d Fumed‐Si‐Pr‐N‐Ter‐Ind: Fumed silica was functionalized with (3‐aminopropyl)trimethoxysilane, terephthalaldehyde, and indandione.

e Fumed‐Silica‐Pr‐TAP: Fumed silica was modified with 3‐(chloropropyl)trimethoxysilane and 2,4,6‐triaminopyrimidine (TAP).

#### 3.2.5 Response Time

**FIGURE 9:**
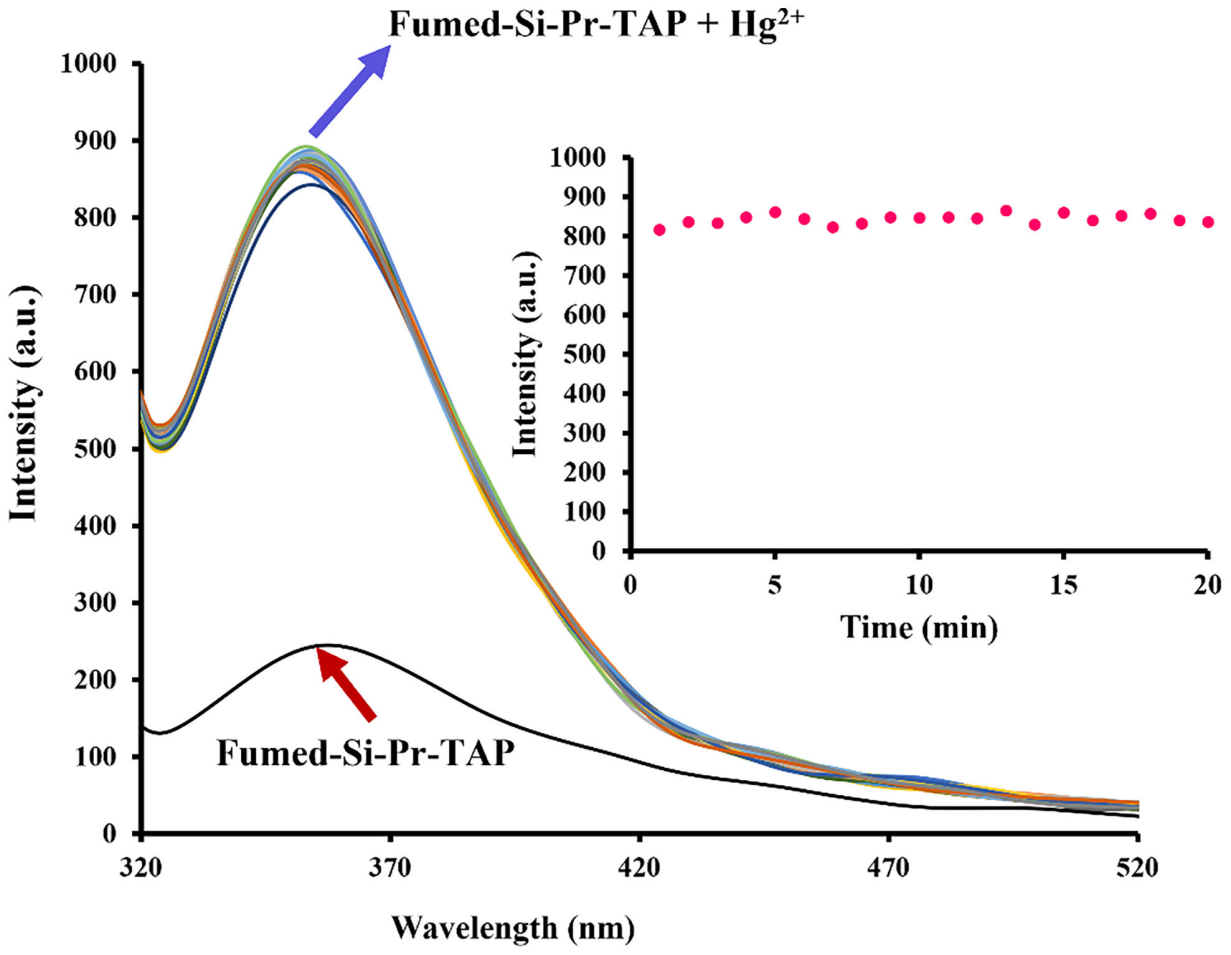
Response time diagram for fumed‐Si‐Pr‐TAP.

The response time of the chemosensor was evaluated over a period of 1–20 min. According to the gained results in Figure 9, it was observed that the emission intensity of the chemosensor in the presence of Hg^2+^ ions did not change significantly over time and had good stability.

#### 3.2.6 pH Effect

**FIGURE 10:**
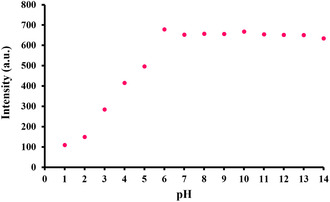
Investigating the effect of pH on the performance of chemosensor fumed‐Si‐Pr‐TAP.

The influence of pH on the response of the fumed‐Si‐Pr‐TAP chemosensor toward Hg^2+^ ions was investigated. As shown in Figure 10, the fluorescence emission intensity increased with increasing pH in the range of 1–6 and attained its maximum value at pH 6. This behavior is due to the protonation of amino groups and nitrogen atoms present in the chemosensor structure in acidic environments, which reduces its ability. At pHs higher than 6, the signal intensity remains almost constant and only minor changes are observed, indicating the stability of the chemosensor binding with Hg^2+^ ions. Therefore, this sensor not only has the highest sensitivity at pH = 6, but also has good performance and applicability in basic environments due to the relative stability of the signal intensity and the lack of significant effect of pH changes on its response.

### 3.3 DFT Perspective

Because the sensing response is primarily governed by the electronic structure and reactive sites of the grafted organic functionality, the theoretical investigation was focused on the organic fragment (Pr‐TAP). The ground‐state geometry of Pr‐TAP was optimized using DFT with the 6‐311 g (d)/LANL2DZ basis‐set combination, and the resulting wavefunctions were analyzed to identify probable interaction sites and relevant quantum‐chemical descriptors.

**FIGURE 11:**
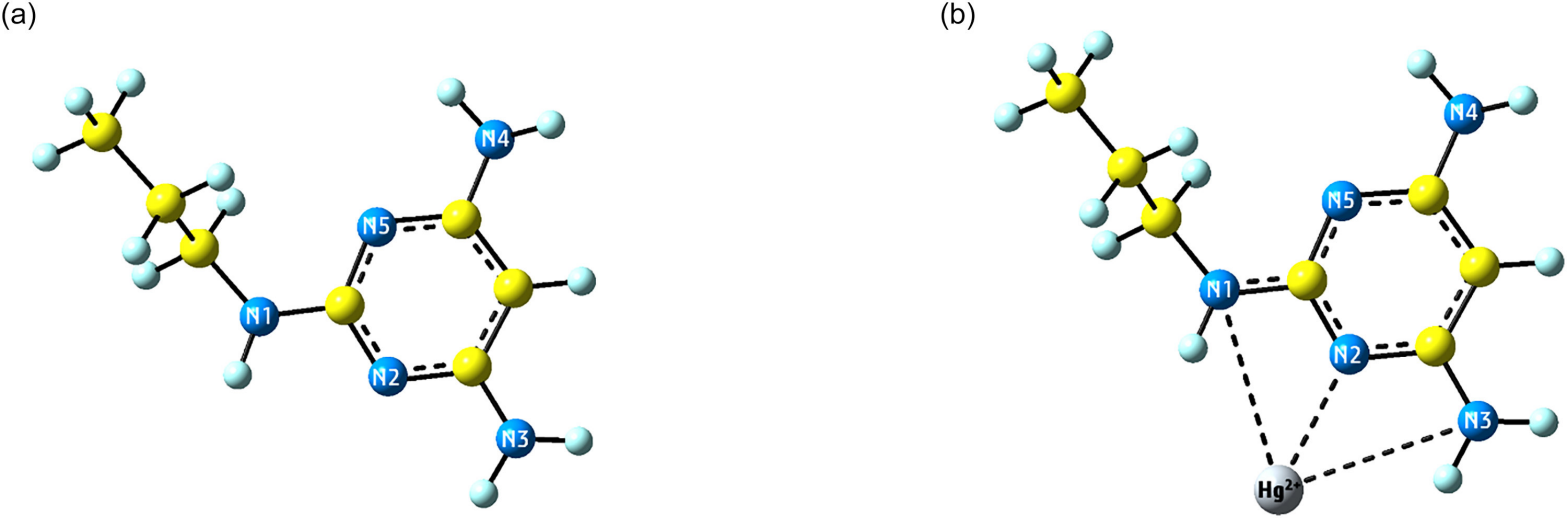
Structure of Pr‐TAP (a) and Pr‐TAP + Hg^2+^ (b) proposed by the DFT perspective.

It should be noted, however, that the silica support was not explicitly included in the computational model. This simplification assumes that the silica surface mainly acts as an inert anchoring matrix and does not directly participate in the electronic interactions responsible for analyte recognition. While this approach enables efficient evaluation of the intrinsic electronic properties of the sensing moiety, it neglects potential surface effects such as local geometric constraints, hydrogen‐bonding interactions, and charge redistribution induced by the silica framework. Consequently, the calculated electronic parameters should be interpreted as representative of the isolated Pr‐TAP fragment rather than the complete surface‐bound system. Nevertheless, the model provides valuable insight into the relative reactivity and probable binding sites of the functional organic unit that governs the sensing behavior. All theoretical data for the optimized structures of Pr‐TAP and the Pr‐TAP + Hg^2+^ complex were obtained from these calculations. The absence of imaginary frequencies confirmed that each optimized structure corresponds to a true minimum on the potential energy surface. The optimized geometries of Pr‐TAP and Pr‐TAP + Hg^2+^ are presented in Figure 11. The Pr‐TAP structure exhibits a minimum energy of −548.49 Hartree. To identify the preferred coordination region, the Hg^2+^ ion was positioned at several possible binding locations around the ligand. The most stable configuration (−591.25 Hartree) was found when Hg^2+^ was coordinated in the region defined by the N1, N2, and N3 atoms, in agreement with the experimentally proposed binding mechanism. To further substantiate this observation, MEP and ESP analyses were carried out.

**FIGURE 12:**
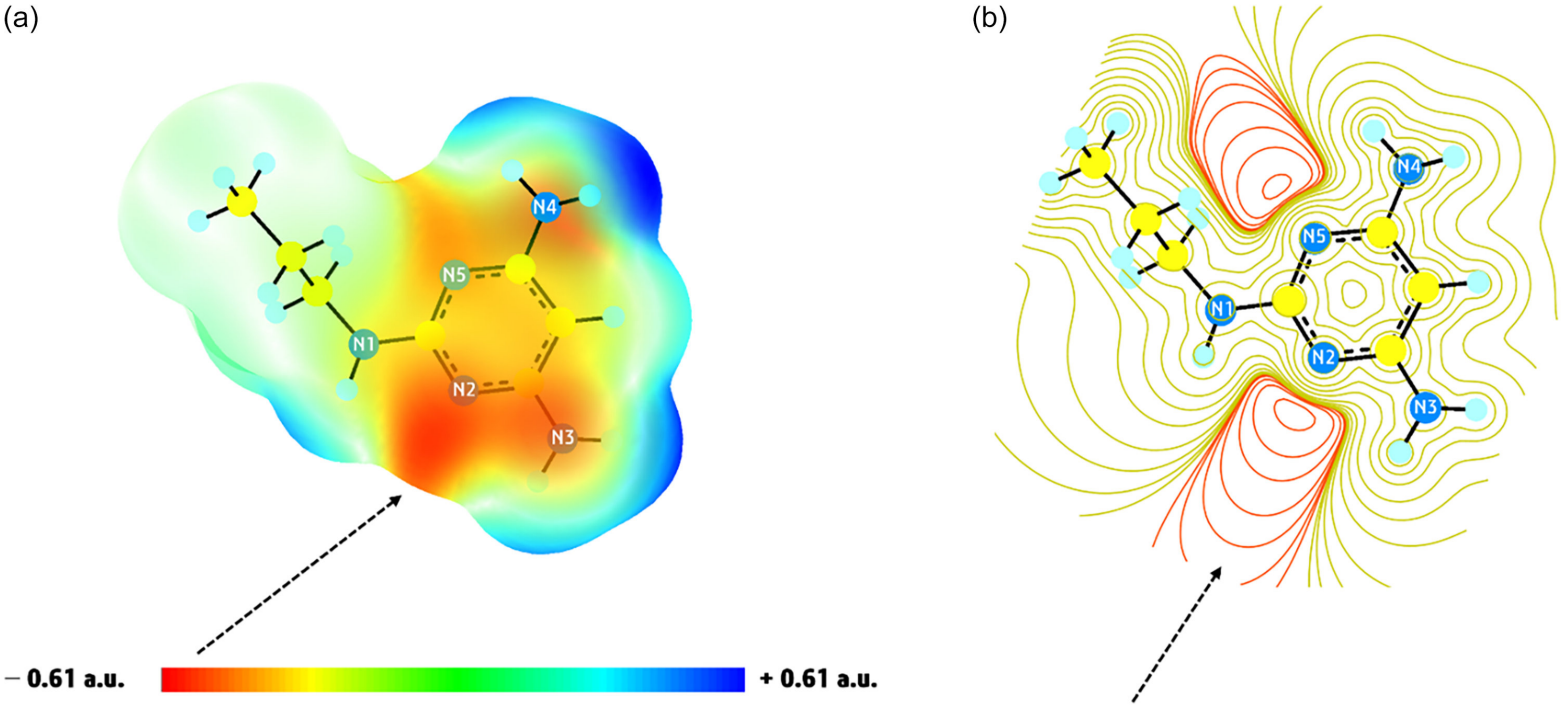
MEP (a) and ESP (b) surface of Pr‐TAP.

The MEP demonstrates the distribution of electronic density across the molecular surface and is widely used to predict reactive sites for electrophilic and nucleophilic processes [48]. Such regions are closely associated with biological activity, hydrogen bonding ability, catalytic behavior, and ion recognition. In an MEP map, electrostatic potential values are represented by color gradients: negative potential is shown in red, whereas positive potential appears in blue. Typically, blue regions are favorable for nucleophilic attack, while red regions favor electrophilic interaction [49]. Electron‐rich red areas, therefore, tend to interact with positively charged species. The MEP surface of Pr‐TAP, calculated from the optimized geometry using the 6‐311 g (d) basis set (Figure 12), reveals blue regions around the hydrogen atoms attached to N1, N3, and N4, suggesting possible sites for nucleophilic attacks. In contrast, pronounced red regions located on electronegative atoms, particularly N2, N3, N4, and N5, indicate higher electron density and stronger attraction toward positively charged ions. Notably, an extended red region delocalized around N2 (between N1, N2, and N3) represents the most favorable coordination site for Hg^2+^ binding. The electrostatic potential ranges from −0.61 a.u. (red) to +0.61 a.u. (blue). Consistently, the ESP map highlights a prominent interaction zone between N1, N2, and N3 atoms. As a result, the DFT‐optimized structures together with MEP and ESP analyses clearly confirm the preferred binding position of mercury in the Pr‐TAP framework, fully supporting the interaction mechanism inferred from the experimental results.

### 3.4 Molecular Orbital Analysis

**FIGURE 13:**
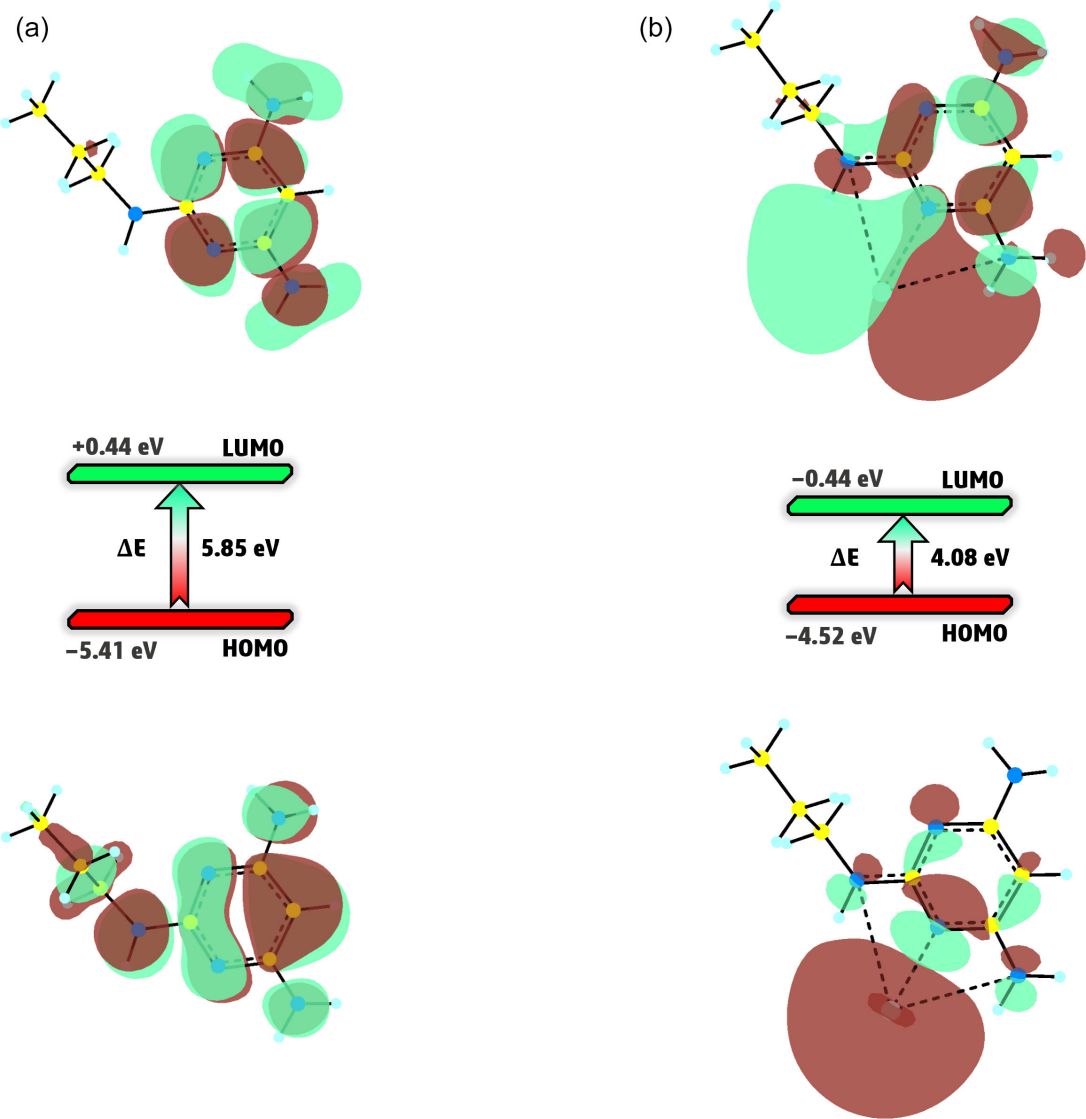
FMOs distribution of (a) Pr‐TAP and (b) Pr‐TAP + Hg^2+^.

The frontier molecular orbitals (FMOs) comprise the HOMO and LUMO. The HOMO is associated with the ionization potential and behaves as an electron donor, whereas the LUMO is related to electron affinity and functions as an electron acceptor [50]. To better elucidate the electron delocalization and sensing performance of Pr‐TAP toward Hg^2+^, molecular orbital calculations were performed within the DFT framework for Pr‐TAP and Pr‐TAP + Hg^2+^. Inspection of the FMO contour plots reveals that, in Pr‐TAP, the HOMO electron density is largely distributed over the entire Pr‐TAP framework, while the LUMO density is predominantly localized on the TAP fragment, suggesting an intramolecular charge transfer from the whole molecule toward the TAP moiety. Upon coordination with Hg^2+^, both the HOMO and LUMO densities become mainly concentrated on the mercury center, as illustrated in Figure 13. The calculated HOMO‐LUMO energy gaps are 5.85 eV for Pr‐TAP and 4.08 eV for Pr‐TAP + Hg^2+^. The reduction in Δ*E* after complex formation indicates enhanced chemical reactivity of the Pr‐TAP in the presence of Hg^2+^. Moreover, the frontier orbital energies in Pr‐TAP + Hg^2+^ lie significantly closer to each other than in the free Pr‐TAP, demonstrating that coordination with Hg^2+^ increases the molecular reactivity by facilitating electronic transitions to higher energy states [51].

## 4 Conclusion

In this study, fumed‐silica was successfully functionalized using 3‐(chloropropyl)triethoxysiliane and TAP agent, and an organic–inorganic hybrid with potential application in fluorescence chemosensors was prepared. SEM images established that the particles had a noticeable change in their shape and overall structure, indicating that the main silica skeleton was maintained after surface modification. Thermal analysis results also confirmed the effective attachment of organic groups to the surface and the appropriate thermal stability of the modified sample. Fluorescence spectroscopy studies showed that this nanostructure exhibits an important and selective response to Hg^2+^ ion, and significant changes in emission intensity are observed in the presence of this ion. The detection limit calculated was 3 × 10^−7^ molar, indicating the appropriate sensitivity of the system in detecting Hg^2+^ ions. Accordingly, fumed‐Si‐Pr‐TAP can be introduced as a selective and efficient fluorescence chemosensor for the detection of Hg^2+^ ions in aqueous environments. The theoretical investigation provides clear evidence that Hg^2+^ preferentially binds within the nitrogen‐containing region of the Pr‐TAP framework, consistent with experimental observations. Electrostatic potential mapping and orbital analysis both point to enhanced electron interaction and redistribution after complexation. The observed decrease in the energy gap further confirms that mercury binding significantly alters the electronic properties of the system, reinforcing the proposed sensing mechanism.

## Author Contributions

**Ghodsi Mohammadi Ziarani:** project administration (lead), supervision (lead), editing (lead). **Fatemeh Soleymani:** writing original draft, doing experiments, and data collection. **Zahra Panahande:** review, editing, validation, software. **Zahra Ahmadi:** doing experiments and data collection. **Alireza Badiei:** software supporting, modifying data. **Senem Akkoc:** validation, software, writing – review and editing. **Mehran Feizi‐Dehnayebi:** writing original draft, review, and editing.

## Funding

The authors have nothing to report.

## Consent

All authors read and approved the final manuscript.

## Conflicts of Interest

The authors declare no conflicts of interest.

## Data Availability

Data will be made obtainable on request to the corresponding author.
